# Elastic microfibril distribution in the cornea: Differences between normal and keratoconic stroma^☆^

**DOI:** 10.1016/j.exer.2017.03.002

**Published:** 2017-06

**Authors:** Tomas L. White, Philip N. Lewis, Robert D. Young, Koji Kitazawa, Tsutomu Inatomi, Shigeru Kinoshita, Keith M. Meek

**Affiliations:** a Structural Biophysics Research Group, School of Optometry and Vision Sciences, Cardiff University, Maindy Road, Cardiff CF24 4HQ, UK; b Department of Ophthalmology, Kyoto Prefectural University, Kyoto, Japan; c Department of Frontier Medical Science and Technology for Ophthalmology, Kyoto Prefectural University, Kyoto, Japan

**Keywords:** Keratoconus, Cornea, Elastic fibers, Fibrillin, Microfibrils, MB, Microfibril bundle, SBF, SEM Serial block face scanning electron microscopy, TEM, Transmission electron microscopy

## Abstract

The optical and biomechanical properties of the cornea are largely governed by the collagen-rich stroma, a layer that represents approximately 90% of the total thickness. Within the stroma, the specific arrangement of superimposed lamellae provides the tissue with tensile strength, whilst the spatial arrangement of individual collagen fibrils within the lamellae confers transparency. In keratoconus, this precise stromal arrangement is lost, resulting in ectasia and visual impairment. In the normal cornea, we previously characterised the three-dimensional arrangement of an elastic fiber network spanning the posterior stroma from limbus-to-limbus. In the peripheral cornea/limbus there are elastin-containing sheets or broad fibers, most of which become microfibril bundles (MBs) with little or no elastin component when reaching the central cornea. The purpose of the current study was to compare this network with the elastic fiber distribution in post-surgical keratoconic corneal buttons, using serial block face scanning electron microscopy and transmission electron microscopy.

We have demonstrated that the MB distribution is very different in keratoconus. MBs are absent from a region of stroma anterior to Descemet's membrane, an area that is densely populated in normal cornea, whilst being concentrated below the epithelium, an area in which they are absent in normal cornea. We contend that these latter microfibrils are produced as a biomechanical response to provide additional strength to the anterior stroma in order to prevent tissue rupture at the apex of the cone. A lack of MBs anterior to Descemet's membrane in keratoconus would alter the biomechanical properties of the tissue, potentially contributing to the pathogenesis of the disease.

## Highlights

• The distribution of microfibril bundles in keratoconus is very different to normal cornea.
• Fibrillin-rich microfibrils in the anterior stroma may be a biomechanical wound response to prevent anterior rupture.
• The absence of an elastic microfibril system in the posterior stroma may contribute to keratoconus pathogenesis.

## 1 Introduction

The transparency and strength of the cornea arise from the highly organised arrangement of the stromal extracellular matrix. The main component of this matrix is type I collagen fibrils that are arranged parallel to each other within lamellae. Corneal transparency is dependent on uniform diameter and quasi-regular spacing between these collagen fibrils (Maurice, 1957), which itself is controlled by the presence of interfibrillar proteoglycans (Kao and Liu, 2003, Lewis et al., 2010). Lamellae are highly interlaced (Radner et al., 1998a) and randomly orientated (Komai and Ushiki, 1991) in the anterior stroma when viewed en face, whereas they are more organised in the posterior stroma, showing two preferred orientations (Abahussin et al., 2009, Aghamohammadzadeh et al., 2004, Meek et al., 1987), and form a circum-corneal annulus at the limbus (Newton and Meek, 1998). This specific arrangement of collagenous lamellae throughout the stroma provides the cornea with the ability to resist tensile strain.

Keratoconus is a pathological condition that is characterised by bilateral progressive stromal thinning and ectasia of the cornea that results in irregular astigmatism and eventual conical shaped cornea as the tissue bows under the influence of intraocular pressure. In more advanced forms of the disease, axial corneal scarring may develop which further reduces the quality of vision. However, the aetiology of keratoconus is currently unclear, as it is likely to involve many different factors including biochemical, genetic and environmental. In advanced stages of keratoconus, the organisation of the collagen lamellae is severely disrupted, resulting in a loss of tensile strength and the progression of ectasia (Daxer and Fratzl, 1997, Meek et al., 2005, Radner et al., 1998b).

As well as collagen, elastic tissue plays a vitally important biomechanical role in many dynamic tissues, for example lungs and blood vessels, allowing them to expand and contract in response to variations in blood pressure (Sherratt, 2009). In similar fashion, the sclera contains an elastic fiber system (Alexander and Garner, 1983, Marshall, 1995) allowing the eye to deform slightly and regain its original shape when impaired by internal and external pressure. Despite the cornea being part of the outer tunic of the eye, along with the sclera, the presence of elastic tissue in the corneal stroma has been overlooked in recent years. Previous studies using histological stains concluded that elastic tissue is absent from mature human cornea (Alexander and Garner, 1983, Hirano et al., 1991), with the exception being Kamma-Lorger et al. (2010), who reported a network of elastic fibers using two-photon fluorescence microscopy.

It is well known that ‘true’ elastic fibers consist of two distinct morphologic components; an outer layer of microfibrils and a central elastin amorphous core (Greenlee et al., 1966, Ross and Bornstein, 1969). Further complexity is added as bundles of microfibrils may exist independently, without an amorphous component; these have historically been termed ‘oxytalan’ (Fullmer and Lillie, 1958). Furthermore, an intermediate fiber containing low quantities of amorphous material has been described, termed ‘elaunin’ (Alexander and Garner, 1983), leading to confusion within the literature in terms of correct terminology.

The development of more advanced imaging techniques, such as serial block face scanning electron microscopy (SBF SEM), has enabled the elastic fiber topic to be re-visited in the cornea. Hanlon et al. (2015) used this technique to demonstrate the presence of ‘elastin-free microfibril bundles’ throughout the murine corneal stroma. These were shown to contain fibrillin-1, a major component of elastic microfibrils (Sakai et al., 1986) but with no observable amorphous elastin core. Furthermore, we have recently characterised a complex elastic system in human cornea using SBF SEM (Lewis et al., 2016). This system consists of elastin-containing sheets at the limbus and narrower micrifibril bundles (MBs) in the central stroma that run parallel to the surface of the cornea, and are highly concentrated in the first ∼10 μm of posterior stroma, immediately anterior to Descemet's membrane. Elastic tissue has previously been reported in various corneal pathologies including bullous keratopathy and Fuchs endothelial dystrophy (Akhtar et al., 2001, Alexander et al., 1981, Ljubimov et al., 1998a). The loss of biomechanical strength and the progression of ectasia in the stroma of keratoconic corneas, as well as the demonstration of an extensive elastic network in normal corneal stroma, highlights the importance of further investigating the presence and distribution of elastic tissue in this disease.

In this paper we used two different elastic fiber-specific staining protocols in combination with SBF SEM and conventional transmission electron microscopy (TEM) to examine elastic tissue throughout the depth of the stroma of keratoconic corneal buttons in comparison to normal cornea, focussing in particular on areas directly anterior to Descemet's membrane, and below the epithelium.

## 2 Methods

### 2.1 Tissue specimens

**Fig. 1:**
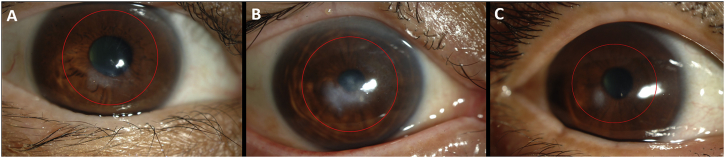
Corneal images in keratoconus before penetrating keratoplasty. Cornea 1 (A) has mild scarring inferior to the pupil, whilst cornea 2 (B) is at a more advanced stage of the disease, hence more severe scarring. Cornea 4 (C) has a small infero-nasal scar. Red circles indicate the regions where 7 mm buttons were taken. (For interpretation of the references to colour in this figure legend, the reader is referred to the web version of this article.)

Four human keratoconus buttons (7 mm diameter) were obtained from the Department of Ophthalmology, Kyoto Prefectural University, Japan, following penetrating keratoplasty. Button 1, from the right eye of a 47-year-old female, had a minor apical scar inferior to the pupil (Fig. 1A). Button 2, obtained from the right eye of a 57-year-old male was more severely scarred in the same region (Fig. 1B). Button 3, from the right eye of a 68-year-old female, was scarred superiorly (no image). Button 4, from the left eye of a 31-year-old male, had a small infero-nasal scar (Fig. 1C). All thinned cone regions were located para-centrally. Following surgery, buttons were immediately fixed in 4% paraformaldehyde before being transported to Cardiff on dry ice. Normal human corneas with scleral rim were obtained from Bristol Eye bank and stored in 4% paraformaldehyde until use. Local ethics committee approval was obtained for this study and the research was carried out in accordance with the tenets of the Declaration of Helsinki.

### 2.2 Serial block face scanning electron microscopy

Thin segments were dissected from each sample and fixed in modified Karnovsky's fixative (2.5% glutaraldehyde and 2% paraformaldehyde in 0.1M cacodylate buffer at pH 7.2) at room temperature before being stored in buffer at 4 °C overnight. Button 1 was processed en bloc using a tannic-acid based staining protocol, whereas buttons 2 and 3 were processed with a novel en bloc orcein staining protocol as below. Normal corneas were processed using both protocols. Sclera control was processed with the orcein protocol only.

Tannic acid based staining protocol: Initially, tissue was processed according to methods described previously (Lewis et al., 2016). Briefly, this consisted of post fixation with 1% osmium tetroxide, incubation in 0.5% tannic acid, staining with 2% aqueous uranyl acetate, dehydration in an ethanol series, further staining with 2% ethanoic uranyl acetate and a saturated solution of lead acetate (Kushida, 1966) before embedding in epoxy resin. This method resulted in dark staining throughout the tissue with little contrast, therefore, additional tissue was processed using an orcein based protocol.

Orcein based staining protocol: Orcein staining has been used to visualise elastic tissue in the cornea using TEM (Lewis et al., 2016), using a protocol based on earlier work (Nakamura et al., 1977). A novel en bloc orcein staining protocol was developed in an attempt to enhance contrast of keratoconic samples using SBF SEM. Samples were fixed with 1% osmium tetroxide before being washed in dH_2_O for 20 min and transferred to 70% ethanol for 10 min. Samples were stained with 0.3% orcein in 70% ethanol for 2 h. After a 30 min wash with 70% ethanol, specimens were dehydrated in 90% ethanol for 20 min, followed by 100% ethanol × 2 for 20 min. Following dehydration, the tissue was subjected to the same remaining steps described in the tannic acid based protocol.

Specimens were examined using a Zeiss Sigma VP FEG SEM equipped with a Gatan 3View2 system, where data sets of up to 1000 images were acquired every 50  nm at 4k × 4k pixel resolution. Three-dimensional reconstructions of data sets were created using Amira 6 software (FEI, Mérignac, France).

### 2.3 Transmission electron microscopy

All corneal samples that were en bloc stained for SBF SEM analysis were also used for TEM. 90 nm gold sections were cut using a Leica UC6 ultra-microtome, floated on distilled water, and mounted on copper grids. All section were visualised using a JEOL 1010 TEM.

## 3 Results

### 3.1 Tannic acid stain

**Fig. 2:**
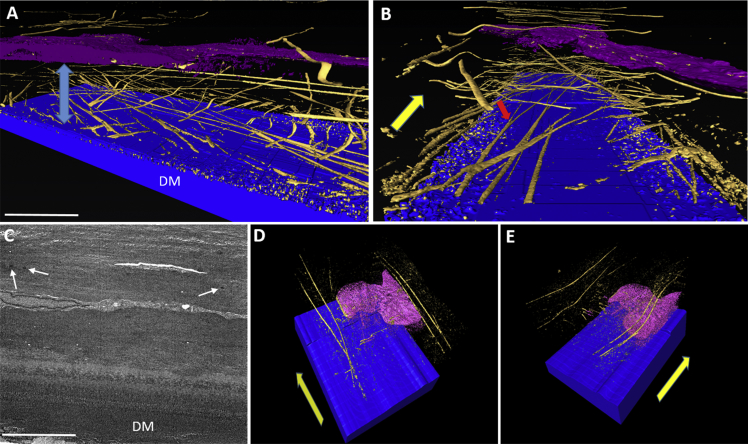
Distribution of MBs anterior to Descemet's membrane (DM) in normal (A–B) and keratoconic button 2 (C–E) corneas, using tannic acid staining. Blue = DM, Gold = MBs, Pink/Purple = Keratocyte. 3D reconstruction in normal cornea (A–B) shows an extensive elastic fiber network concentrated between DM and the keratocyte (blue arrow). These structures run towards the limbus (yellow arrow), parallel to the surface of the cornea and were seen to bifurcate (red arrow). SBF SEM images from keratoconic cornea displayed poor contrast (C), although a small number of electron dense MBs were visible (white arrows). 3D rendering of this data set shows four MBs running longitudinally, anterior to the keratocyte, towards the limbus (D–E) (yellow arrows). No MBs were present directly anterior to DM. Scale bar = 5 μm. (For interpretation of the references to colour in this figure legend, the reader is referred to the web version of this article.)

Normal cornea stained with tannic acid displayed an extensive MB system in the central and peripheral cornea, concentrated immediately anterior to Descemet's membrane (Fig. 2A–B). These MBs ran transversely and longitudinally, parallel to the plane of the cornea, from limbus to limbus, and were seen to bifurcate and trifurcate. SBF SEM data sets obtained from regions of stroma overlying Descemet's membrane in keratoconic button 1 appeared very dark with little contrast, so it appeared that no MBs were present, and therefore, it was difficult to produce 3D reconstructions. However, one data set produced visible MBs that were rendered to produce a 3D reconstruction (Fig. 2C–E). The lack of contrast is evident as it is difficult to distinguish between Descemet's membrane and posterior stroma, a junction that is clearly evident in normal cornea using the same staining protocol. Image reconstruction of the keratoconic data set revealed four MBs lying anterior to the deepest stromal keratocyte, running in the same direction, parallel to the surface of the cornea and towards the limbus. No MBs were visible in the stromal space between the keratocyte and Descemet's membrane. The profound difference in MB concentration between normal and keratoconic cornea was clearly evident. No MB stain was visible in the ∼10 μm region of stroma directly anterior to Descemet's membrane in keratoconic cornea, compared to the extensive network of MBs observed in normal cornea.

In the SBF SEM data set obtained from a sub-epithelial location in keratoconic cornea, a ∼5 μm band of dark staining was observed. The MBs appeared very small, and therefore they were difficult to clearly distinguish with the lower resolution imaging of SEM. This, in combination with poor contrast meant that it was not possible to transform the data set into a 3D reconstruction. A single image from the data set is shown in Fig. 4E.

**Fig. 3:**
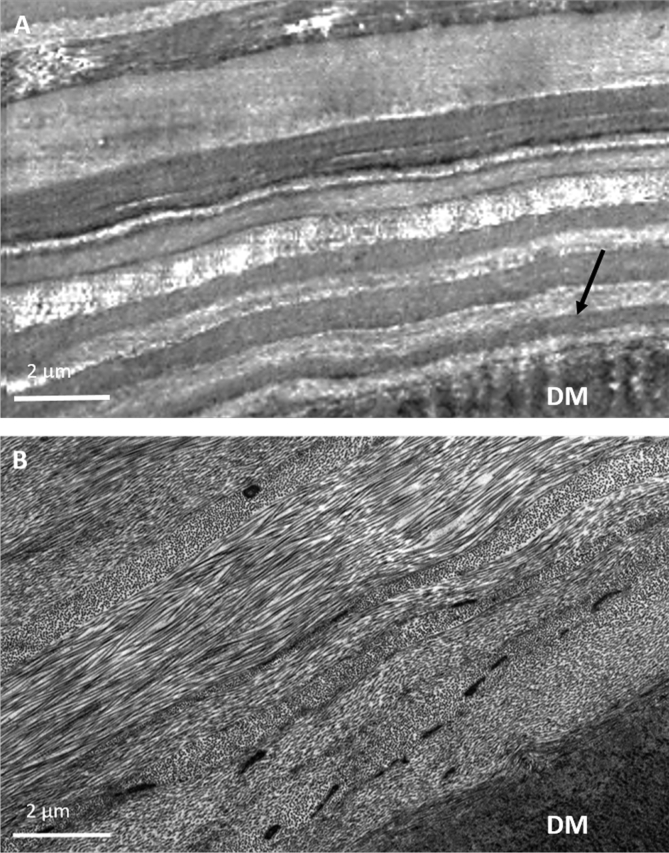
The posterior stroma and Descemet's membrane (DM) stained with tannic acid. In keratoconic button 1 cornea (A), the TEM image shows the first ∼10 μm of stroma and reveals no MBs in the stroma (arrow) above Descemet's membrane. Conversely, in the same region of normal cornea (B), an abundance of electron dense MBs are visible in the first ∼8 μm of stroma above DM.

**Fig. 4:**
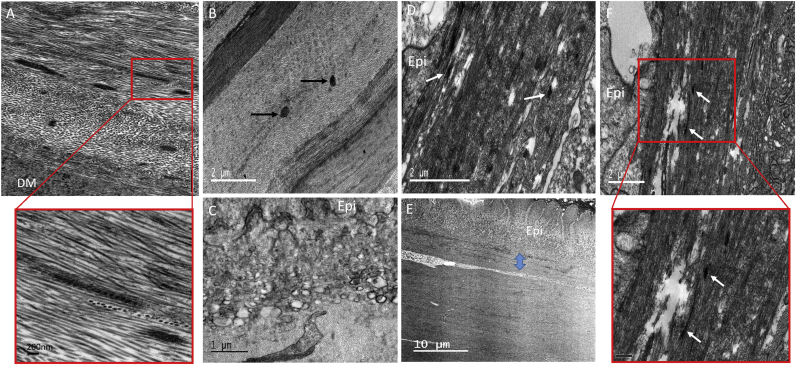
TEM micrographs of normal (A) and keratoconic button 1 (B–F) cornea using tannic acid staining. Normal cornea contains a concentration of MBs anterior to DM (A), with longitudinally sectioned structures showing a ∼60 nm periodicity. In keratoconic cornea (B), a small number of MBs were located in the posterior and middle stroma after extensive searching (black arrows). Many vacuoles were discovered at the border between basal epithelium (Epi) and stroma (C) with the absence of basal lamina and Bowman's layer. Many MBs were seen directly below the basal epithelium (white arrows) (D–F), concentrated in a 5 μm region (blue arrow). These were oriented longitudinally and transversely (inset – scale bar 0.5 μm). (For interpretation of the references to colour in this figure legend, the reader is referred to the web version of this article.)

TEM provided clearer images of keratoconic cornea with better contrast compared to SEM. When viewing the area of stroma anterior to Descemet's membrane, it was difficult to detect any MBs in the center or periphery of the button (Fig. 3A), compared to the concentration of MBs seen in the same area of normal cornea (Fig. 3B). Viewing these structures in the normal cornea at high magnification revealed a periodic banding pattern repeating every 50–60 nm (Fig. 4A inset). Few MBs could be seen after an extensive search of the stroma in both keratoconic buttons 2 and 3; these were sectioned transversely, with a diameter of ∼250 nm (Fig. 4B). Many vacuoles were seen protruding from some areas of epithelium in keratoconic cornea, with the absence of basal lamina and Bowman's layer (Fig. 4C). Furthermore, an abundance of electron dense MBs were discovered between the basal epithelium and stroma (Fig. 4D–F). These were orientated both transversely and longitudinally, running along the plane of the cornea. In the same area, breaks in the stroma were evident.

### 3.2 Orcein stain

**Fig. 5:**
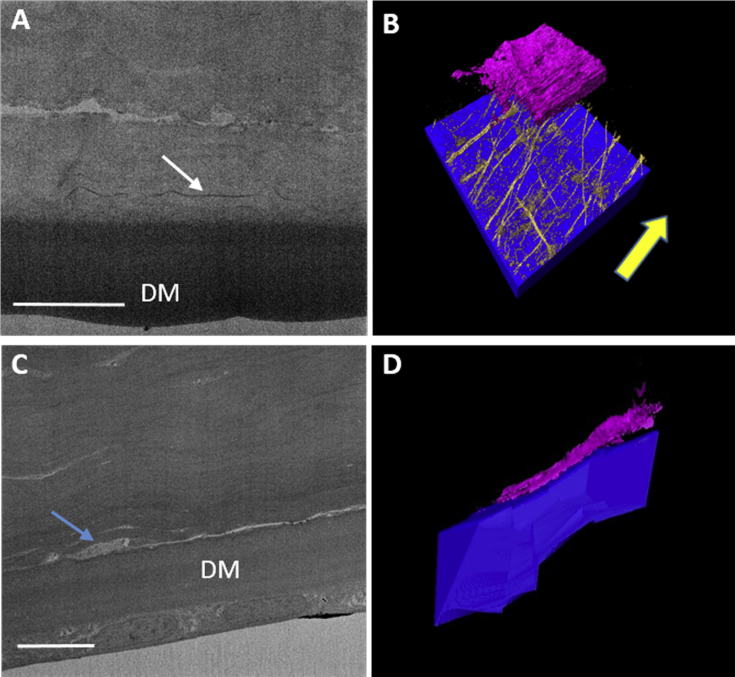
Distribution of MBs anterior to Descemet's membrane (DM) in normal (A–B) and keratoconic button 2 (C–D) cornea using orcein staining. Blue = DM, Gold = MBs, Pink/Purple = Keratocyte. Densely-stained MBs (white arrow) seen anterior to DM in normal cornea (A) were reconstructed, resulting in a 3D model (B) where the MBs were similar in number and orientation to those we have previously described in tannic acid stained cornea, with the majority travelling in one direction (yellow arrow). No MBs were identified in the keratoconic cornea data set, with poor contrast between DM and stroma (C). A keratocyte (blue arrow) was seen lying directly on top of DM (C–D), a finding that was seen frequently in other keratoconic data sets. Scale bars = 10 μm. (For interpretation of the references to colour in this figure legend, the reader is referred to the web version of this article.)

An attempt was made to improve MB image contrast by removing uranyl acetate, which predominately stains collagen, and replace it with the well-known elastic fiber stain, orcein. Despite this change, data sets obtained from keratoconic buttons 2 and 3 still appeared to lack contrast, making it difficult to produce 3D reconstructions. A single image from the data set acquired from the normal cornea stained with orcein shows good contrast between Descemet's membrane and overlying stroma, and electron dense MBs can easily be identified in this region (Fig. 5A). Reconstructing this data set showed a concentration of electron dense MBs lying directly anterior to Descemet's membrane (Fig. 5B) that were very similar in concentration and orientation to those seen using the tannic acid stain. This provided evidence that both protocols are staining the same structures. The majority of these MBs ran longitudinally towards the limbus, with some travelling transversely. No MBs were identified in the 3D reconstructions anterior to Descemet's membrane in keratoconic cornea (Fig. 5C–D), although this may have been due to the poor contrast and image quality, as mentioned previously. Throughout many keratoconus data sets, many keratocytes were seen lying directly anterior to Descemet's membrane (Fig. 5D), something rarely observed in normal cornea.

**Fig. 6:**
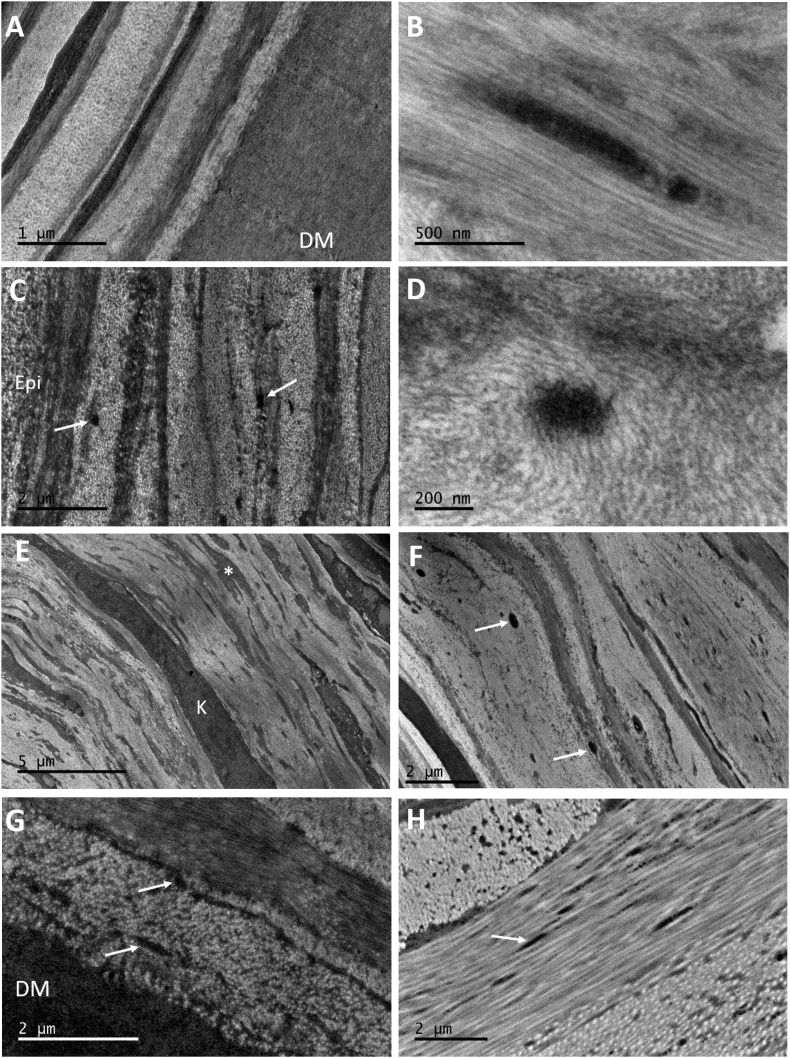
TEM micrographs of orcein stained keratoconic cornea buttons 2 and 3 (A–F) and normal controls (G–H), showing the presence of MBs (white arrows). No MBs are seen anterior to DM (A). MBs were occasionally seen in the posterior and mid stroma, with longitudinally sectioned structures displaying a faint ∼50–60 nm periodicity characteristic of fibrillin-1 (B). MBs were concentrated in the anterior stroma, below the epithelium (C). (A full three-dimensional structure from button 4 is seen in video clip S1). High magnification images of transverse MBs show the presence of individual microfibrils within the bundle (D). Scarred areas of stroma contained a mass of electron dense material (asterisk) (E) and an abundance of MBs (button 2) (F). MBs were seen in normal control tissue – cornea (G) and sclera (H).

As with SEM, TEM results from keratoconic buttons 2 and 3 stained with orcein were consistent with those from the tannic acid staining protocol. MBs were sparsely populated anterior to Descemet's membrane (Fig. 6A), with only the occasional bundle visible in which the characteristic 50–60 nm periodicity was difficult to discern (Fig. 6B). Similar to the tannic acid-stained keratoconic button 1, electron dense MBs were visualised in abundance below the epithelium, with diameters ranging from 100 to 200 nm (Fig. 6C). These structures were seen both between the basal lamina and the intact Bowman's layer, and also directly posterior to Bowman's layer. By viewing these structures at a high magnification, the individual microfibrils making up the bundles could be seen (Fig. 6D). The diameter of these individual microfibrils appeared to be in the range 7–13 nm.

Supplementary video related to this article can be found at http://dx.doi.org/10.1016/j.exer.2017.03.002

The following is/are the supplementary data related to this article:

Video 1 Rendered three-dimensional video of microfibril bundles in the anterior stroma of cornea button 4. The MBs are very thin and difficult to segment away from the noisy background, but they are clearly visible running in planes roughly parallel to the corneal surface. Video1

Scarred areas of tissue seen in keratoconic button 2 were also examined using TEM. Electron-dense material surrounded keratocytes/fibroblasts throughout the entire stroma of the scarred tissue (Fig. 6E). In some areas of scar tissue, large amounts of densely-stained MBs were seen, with diameters ranging from 70 to 300 nm (Fig. 6F). Normal cornea and sclera were used as controls for the orcein stain, with both demonstrating the presence of MBs (Fig. 6G–H).

## 4 Discussion

Both the tannic acid (Kageyama et al., 1985, Simmons and Avery, 1980) and orcein-based (Nakamura et al., 1977) protocols are known to stain the microfibrillar and amorphous components of elastic fibers. When applied to keratoconic corneas, SBF SEM revealed poor image contrast, although it was still possible to identify MBs where present. It is not clear why processed keratoconic buttons produced poor image contrast as the same protocol worked very well for human (Lewis et al., 2016), porcine, and murine cornea (White et al., 2017). Nevertheless, using both staining protocols, this study has shown that the presence of MBs in the central stroma of keratoconic cornea is very different to the normal healthy cornea.

SBF SEM volume reconstructions in the area of stroma immediately anterior to Descemet's membrane showed a significant difference in MB concentration in keratoconic cornea compared to normal cornea. Both staining protocols clearly demonstrated the presence of an extensive network of MBs anterior to Descemet's membrane in normal cornea, as we have previously described (Lewis et al., 2016). Many data sets were obtained from the same area, at all radial positions, from both tannic acid and orcein stained keratoconic buttons, with MBs visible in only one reconstruction. The lack of electron-dense structures in this region was confirmed with TEM, where an extensive search was required to identify any MBs in this area. Longitudinally orientated MBs displayed a banding pattern with periodicity of 50–60 nm, characteristic of fibrillin-1 containing microfibrils (Jensen et al., 2012, Sherratt et al., 2003), which was clearer in normal cornea. Frequently, keratocytes were seen lying directly opposed to Descemet's membrane, in a ‘pre-Descemet's layer’ of stroma that Dua et al. (2013) described as acellular in normal cornea, although this has been disputed (Jester et al., 2013, Schlotzer-Schrehardt et al., 2015). This evidence implies that pre-Descemet's layer may not exist in keratoconic cornea. As the samples were corneal buttons, it is unknown if there is a lack of elastic tissue at the periphery and limbus of the keratoconic cornea.

**Fig. 7:**
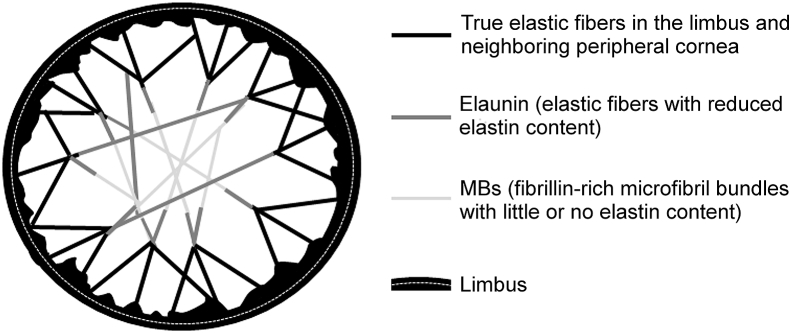
Schematic diagram of the proposed elastic fiber network in the corneal stroma. Elastic fibers consisting of predominantly fibrillin microfibrils sheathing an amorphous elastin core occur pseudo-circumferentially in the limbus and become more like sheets, then broad fibers towards the cornea (black lines). As the fibers continue towards the center of the stroma, they become thinner and start to lose their elastic cores (grey). In the central optical zone of the cornea, there is little or no elastin and most fibers become fibrillin-rich bundles of microfibrils (pale grey). In the keratoconus central cornea, the MBs are greatly depleted so the potential connections across the cornea are reduced and, we propose, the cornea is subsequently weakened.

**Supplementary Fig. S1:**
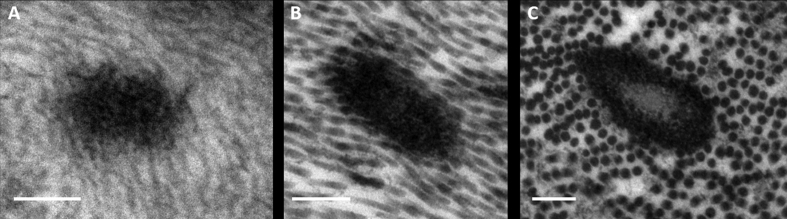
High magnification TEM images of elastic material in human cornea. When viewed in cross-section, elastic material in central keratoconic (A) and normal (B) cornea appears as electron-dense MBs, whereas at the limbus of normal cornea (C), elastic material consists of a central amorphous component surrounded by electron-dense microfibrils. Scale bars = 200 nm.

Our previous three-dimensional SBF SEM study (Lewis et al., 2016) showed that normal human cornea contains a complex system of elastic fibers that run from limbus to limbus. The suggestion from this work was that the nature of these fibers varies in different regions of the cornea. In the limbus they occur as reticulated sheets and are thus true elastic fibers with an amorphous elastin core (supplemental Figure S1). We propose that, as they progress through the peripheral cornea, they lose some of the amorphous elastin component within the core and become what Alexander and Garner (1983) referred to as elaunan. By the time they reach the central cornea, the majority have lost most, if not all, of the amorphous elastin, and become MBs (oxytalan) (Fig. 7).

Such a model would have practical biomechanical implications. During the intraocular pulse, the cornea moves forward, but the optically important central cornea retains its shape, with deformation mostly in the peripheral cornea and limbus (Boyce et al., 2008). This would be facilitated by a system such as we propose here, in which the extensibility of the elastic fiber system is greatest in the periphery and less in the center, but which retains the need for the fibers to be clamped at each end in order to transmit the loads across the cornea. Whereas the elastin component of elastic fibers provides tissue with elasticity, fibrillin-1 microfibrils primarily provide rigidity and act to reinforce the elastic fiber (Sherratt et al., 2003).

MBs are present in foetal cornea (Lewis et al., 2016) and in the posterior stroma of young infants (Alexander and Garner, 1983), therefore, patients with keratoconic corneas are either born with a lack of elastic material in this region, or it is degraded throughout life. MBs are important in the development of tissues containing elastic material, as they provide a scaffold for the deposition of elastin during elastogenesis (Jones et al., 1980, Kewley et al., 1978). They also link elastic fibers to each other (Fig. 7) and to other components of the extracellular matrix, and thus play a role in tissue homeostasis by engaging in various cell-matrix interactions (Jensen and Handford, 2016, Kielty and Shuttleworth, 1995). In other tissues, the importance of MBs in maintaining tissue function is highlighted in Marfan syndrome, an autosomal dominant disease caused by defects in the *FBN1* gene (Dietz et al., 1991), leading to a lack of/disorganised elastic tissue in the extracellular matrix, resulting in cardiovascular, skeletal, and ocular abnormalities. This disease often leads to features that overlap with keratoconus including astigmatism, corneal thinning, and flattened cornea (Heur et al., 2008, Konradsen et al., 2012, Sultan et al., 2002). An example of the importance of the biomechanical strength conferred by MBs is evident in ciliary zonules, where an abundance of fibrillin-1 microfibrils is responsible for holding the lens in dynamic suspension, with a loss of this structural anchorage in Marfan syndrome leading to ectopia lentis (Ashworth et al., 2000). Differences observed in MB distribution between the stroma of normal and keratoconic cornea would thus be expected to alter the biomechanical properties of the tissue. In terms of degradation, it is thought that collagen is broken down by proteolytic enzymes in keratoconus, resulting in corneal thinning (Balasubramanian et al., 2010). Fibrillin-1 domain structure is dominated by cbEGF (calcium binding epidermal growth factor) domains, where calcium binding is thought to rigidify the microfibrils and protect them from proteolysis (Reinhardt et al., 1997). Lysyl oxidase activity, an enzyme involved in cross linking, is decreased in the stroma of keratoconus corneas (Dudakova et al., 2012). Furthermore, lower levels of lysinonorleucine, a lysl oxidase-derived crosslink found in collagen and elastic fibers, are reported in the stroma of keratoconus cornea (Takaoka et al., 2016), meaning a loss of strength and stability of elastic fibers, which could subsequently render them more susceptible to degradation.

In contrast to the posterior stroma, MBs were concentrated in a ∼5 μm band in the anterior stroma below the epithelium in thinned central regions of keratoconus buttons, as observed by Jefferies and Alexander (1995) with light microscopy. This is a finding that was not seen in full thickness quantification analysis of normal cornea, where no elastic material was detected below the epithelium (Lewis et al., 2016). These MBs were much smaller in diameter when compared to those seen in the middle and posterior stroma; this, along with poor contrast, made it difficult to create the 3D reconstruction (Video clip S1).

It is thought that the corneal thinning observed in keratoconus is a result of a loss of stromal tissue (Mathew et al., 2011, Morishige et al., 2007), although the precise mechanisms leading to this are currently unknown. In the normal cornea, the anterior stroma is believed to be biomechanically stronger than the posterior stroma due to a high degree of lamellar interweaving (Komai and Ushiki, 1991, Morishige et al., 2006); however, numerous studies have shown that lamellar interweaving is significantly reduced in the anterior stroma of keratoconus corneas, as well as a loss of collagen lamellae inserting into Bowman's layer (Mathew et al., 2015, Morishige et al., 2007, Morishige et al., 2014, Radner et al., 1998a), ultimately facilitating lamellar “slippage” (Meek et al., 2005). The central anterior cornea is a weak area in keratoconus, forming the apex of the cone, and likely in need of additional biomechanical strength to that provided by collagen. An increased concentration of MBs below the epithelium/Bowman's layer in keratoconus corneas may be a wound repair response to biomechanically stabilise this weakened area to prevent rupture. If this is correct, the presence of MBs in this area, which were not observed in normal cornea (Lewis et al., 2016), may also have an effect on the shape of the cornea and could potentially contribute to cone formation.

Abnormalities in Bowman's layer were observed, along with the presence of vacuoles, suggesting that proteases are being released from a degenerating basal epithelium. In an early electron microscopy study, Teng (1963) suggested that one of the earliest microscopical changes observed in keratoconus is degeneration of epithelial cells, with simultaneous release of unspecified enzymes, disrupting Bowman's layer and ultimately resulting in anterior stromal scarring. This may be occurring in button 1, although scarring did not reach the level of severity seen in button 2, which was at a more advanced stage of the disease. The areas of stroma with scarring present were very different, with the majority consisting of disorganised collagen and an abundance of electron dense material, as previously observed in the stroma of keratoconus tissue (Pouliquen, 1987). In addition to this, a concentration of structures with a very similar appearance to MBs were identified throughout the scarred stroma. Newly synthesised elastic fibers have been reported in scar tissue of skin (Roten et al., 1996, Tsuji and Sawabe, 1987), but little work has been carried out in the cornea. Kenney et al. (1997) discovered fibrillin-1 in fibrotic regions of keratoconic corneas, whereas Ljubimov et al. (1998b) described abnormal deposits of fibrillin-1 in stromal scars after radial keratotomy. Fibrillin-1 labelling in these scars is likely to be caused by the MBs we describe in this study. It is likely that this material is laid down by fibroblasts, along with collagen and other extracellular material, in order to strengthen the repairing stroma.

## 5 Conclusion

In conclusion, the distribution of MBs in the stroma of keratoconus is very different to normal cornea. The stroma of normal cornea contains a network of MBs anterior to Descemet's membrane, becoming progressively less abundant anteriorly, with none detected below the epithelium. This finding is reversed in non-scarred regions of keratoconus, with a concentration of MBs below the basal epithelium in thinned central regions, and very few anterior to Descemet's membrane and throughout the rest of the stroma. It is likely that these structures are produced to provide additional biomechanical stability to the anterior stroma in order to prevent tissue rupture at the apex of the cone. A lack of an elastic system anterior to Descemet's membrane in keratoconus would render this area biomechanically less responsive than normal cornea, potentially contributing to the pathogenesis of the disease.

## Conflict of interest

No conflicting relationship exists for any author.
