# Supplementary figures and images for: Elastic microfibril distribution in the cornea: Differences between normal and keratoconic stroma
Source: Exp Eye Res. 2017 Jun;159:40–8. doi: 10.1016/j.exer.2017.03.002 (PMC5451143; doi:10.1016/j.exer.2017.03.002)

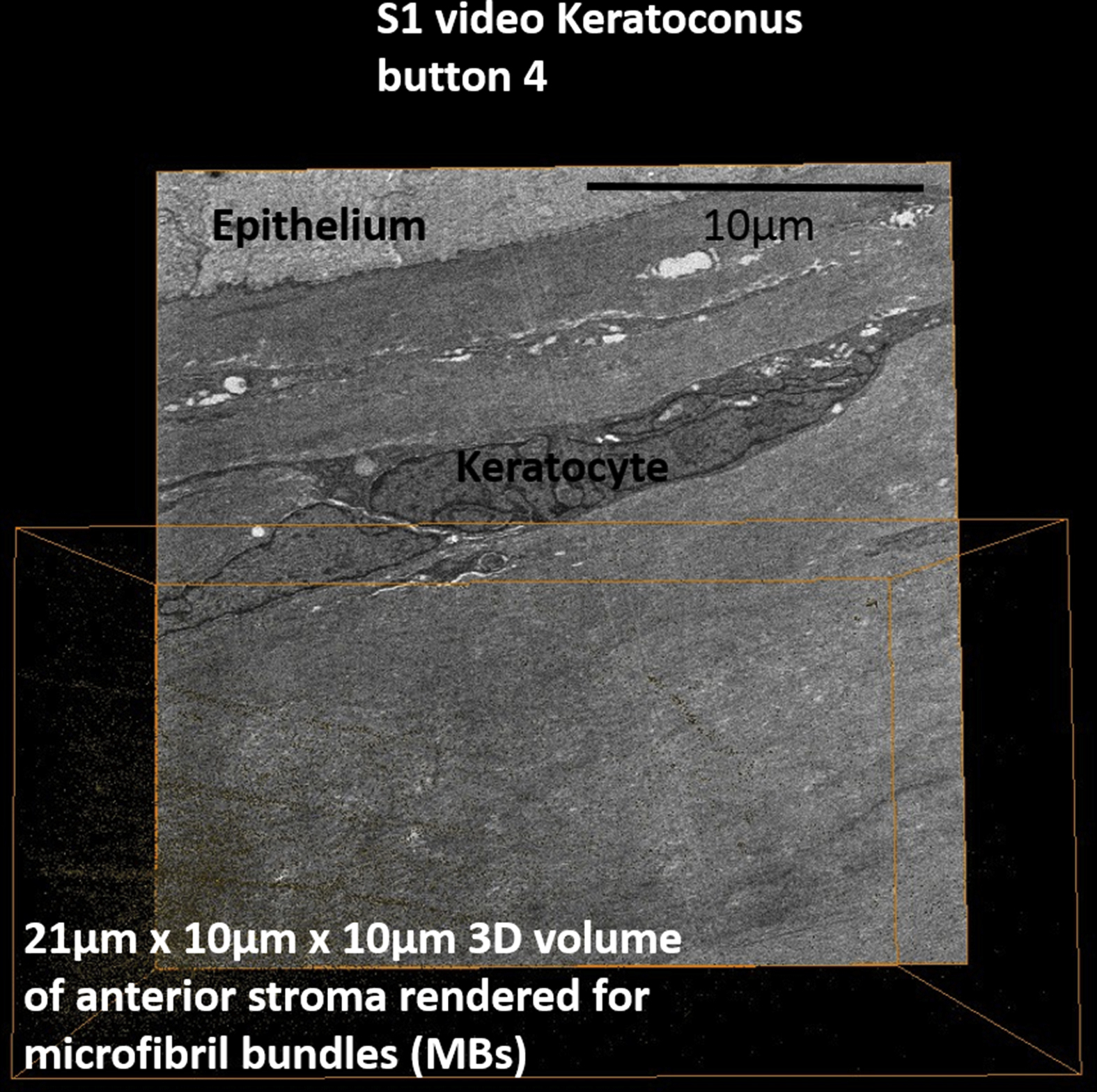

Supplement: Video 1 — Rendered three-dimensional video of microfibril bundles in the anterior stroma of cornea button 4. The MBs are very thin and difficult to segment away from the noisy background, but they are clearly visible running in planes roughly parallel to the corneal surface. [file mmc1.jpg]
